# Health horizons: Future trends and technologies from the European Medicines Agency’s horizon scanning collaborations

**DOI:** 10.3389/fmed.2022.1064003

**Published:** 2022-12-08

**Authors:** Valentina Vignali, Philip A. Hines, Ana Glória Cruz, Barbara Ziętek, Ralf Herold

**Affiliations:** ^1^European Medicines Agency, Amsterdam, Netherlands; ^2^Department of Biomedical Engineering, W.J. Kolff Institute, University Medical Center Groningen, Groningen, Netherlands; ^3^Faculty of Health Medicines and Life Sciences, Maastricht University, Maastricht, Netherlands

**Keywords:** horizon scanning, medicines regulation, public health, innovation, preparedness, health technology

## Abstract

In medicines development, the progress in science and technology is accelerating. Awareness of these developments and their associated challenges and opportunities is essential for medicines regulators and others to translate them into benefits for society. In this context, the European Medicines Agency uses horizon scanning to shine a light on early signals of relevant innovation and technological trends with impact on medicinal products. This article provides the results of systematic horizon scanning exercises conducted by the Agency, in collaboration with the World Health Organization (WHO) and the European Commission’s Joint Research Centre’s (DG JRC). These collaborative exercises aim to inform policy-makers of new trends and increase preparedness in responding to them. A subset of 25 technological trends, divided into three clusters were selected and reviewed from the perspective of medicines regulators. For each of these trends, the expected impact and challenges for their adoption are discussed, along with recommendations for developers, regulators and policy makers.

## Introduction

Societal progress is enabling an increasing number of scientific breakthroughs across multiple sectors, many of which have ramifications beyond their sector of origin. For example, economic development is driving forward research and development in areas such as biomedicine and computing, enabling bioinformatics and subsequent discovery of new medicinal products. For society to benefit to the greatest extent from these innovations, however, the implementation of relevant technologies needs to be facilitated, following an utility evaluation and impact analysis.

This is especially true in medicines development, where the journey from discovery to application is finely regulated, and efficient interactions among multiple stakeholders are crucial for new technologies to be adopted (1).

To make the most of this rapid pace of advancement, in addition to the medicine regulators’ gatekeeping role safeguarding medicines in the EU, they have an increasing role as facilitators or enablers of innovation, providing guidelines and scientific advice to developers prior to the evaluation of marketing authorization applications. Moreover, as highlighted by the COVID-19 pandemic, immediate and coordinated regulatory actions have been fundamental for the safe and rapid implementation of novel solutions during a crisis (2, 3). Hence the need for regulatory authorities to not only keep pace with innovation and facilitate opportunities but also to monitor potential future threats *via* appropriate foresight methodologies.

Horizon scanning is a powerful research tool for identifying, prioritizing and assessing signals of novel developments and issues concerning public health, which could have an impact in the next 3–10 years (4, 5).

Therefore, the European Medicines Agency (EMA) adopted horizon scanning as a method for future-proofing and is actively involved in monitoring activities aimed at strengthening the European Regulatory Network preparedness.

In this research article, the outcome of horizon scanning exercises conducted by the EMA in collaboration with World Health Organization (WHO) and the European Commission’s Directorate-General Joint Research Centre (JRC) are reported. The two different modalities of signal identification allowed a broad coverage of the innovation and public health landscape.

The strategy used by the WHO’s exercise consisted in a top-down approach led by experts including EU regulators, while the JRC adopted a bottom-up bibliometric method to which EMA contributed, providing domain knowledge for specific sectors (6, 7).

The most impactful topics for medicines development identified using both approaches are discussed and analyzed in this publication. For each signal, the maturity of the technology or trend and its potential impact on medicines regulation and public health was assessed.

This research demonstrates the value of collaborative, structured foresight activities. The results obtained will be used to improve preparedness of EMA to future technologies and trends. In addition, this document provides awareness of these technologies or trends, and their challenges and opportunities, which have relevance for all stakeholders involved in healthcare innovation and decision-making.

## Materials and methods

### Signal identification

**FIGURE 1:**
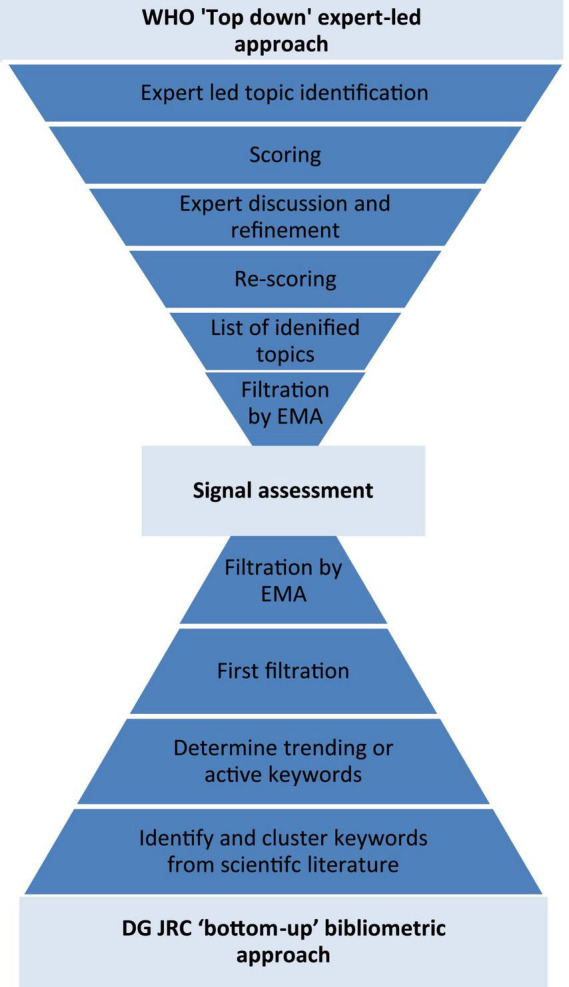
Flow diagram of the two methods to identify signals: the WHO “top-down” expert provision of signals, and the DG JRC “bottom-up” bibliometric analysis.

The horizon scanning to identify signals was done using two approaches: “top-down” expert provision of signals, and a “bottom-up” bibliometric analysis to determine signals (Figure 1).

The “top-down” expert-led approach was undertaken by the World Health Organization in their “Emerging trends and technologies: a horizon scan for global public health” exercise (6). This exercise applied a mixed qualitative and quantitative method to elucidate signals from experts: “investigate, discuss, estimate, aggregate (IDEA)” protocol (6, 8). Here health experts, including from the EMA and its network, self-identified signals “that will shape the future of global health”; before scoring them according to their impact, plausibility and novelty. The highest scoring of these signals was then elaborated on by the expert participants before being discussed in an online, group setting. After the discussion the signals were re-scored and the discussion used to create a final ranking and assessment of the signals.

The “bottom-up” bibliometric approach was undertaken by European Commission’s Joint Research Centre’s (DG JRC) in their “Weak signals in Science and Technologies–Weak signals in 2020” exercise (7). The DG JRC’s approach used text mining to identify and cluster keywords from the Scopus database (2016–2020), resulting in more than 4 million clusters. The keyword clusters were then used to search Scopus from 1996 to 2020 and the number of recent publications containing these keywords clusters were compared with past publications to quantify how novel or “active” the keywords or “signals” are. Those signals which were very “active” were kept and filtered further by a minimum number of documents within each cluster, the “semantic compactness” between the publications within the cluster, and a variety of custom filters (7). Finally, the DG JRC’s TIM Technology system, containing scientific publications, patents and EU R&D grants, was used to maximize the information available about each signal for filtration and assessment by DG JRC and EMA experts.

### Signal filtration

The signals from the above two exercises were then screened by the authors using the following qualitative inclusion criteria based on their potential impact. These criteria were derived from a previous systematic literature review into Horizon Scanning methods (4):

• Likely to impact medicines development within the next 10 years

AND potential impact on EMA’s:

• Ability to assess efficacy of medicinal products OR;
• Ability to assess safety issues of medicinal products/technologies/methodologies OR;
• Ability to assess quality of medicinal products;

OR the signal poses substantial:

• Ethical challenges OR;
• Legal challenges OR;
• Level of novelty OR;
• Potential to address a high unmet medical need.

Signals of individual commercial developments were excluded from this publication to avoid the perception or prejudice of regulatory opinions. Signals of duplicate topics were either removed or elements converged.

### Signal assessment

The chosen signals were then analysed by the authors for their potential impact as per the above criteria. In addition, an analysis of how EMA should respond to them was initiated in parallel. Follow-up actions may be disclosed in due time. This analysis was then peer-reviewed by EMA subject matter experts.

## Results

### World Health Organization horizon scanning

The topics below were derived from the 58 long-list of signals from the WHO’s horizon scanning exercise (6).

#### The internet of medical things

The Internet of Medical Things (IoMT) is a communication environment that connects medical devices, software applications, health systems and services. It has the potential to improve care for patients in a variety of ways and facilitate personalized medicines application. The data collection possible through the IoMT can enable early diagnoses and treatment. It also allows remote patient monitoring and care, reducing the burden on health care systems and the environmental impact of the need to travel, improve care for remote communities and overall improving patient experiences (9).

Advances in computing, wireless capabilities (5G) and biomedical devices, combined with the recent solutions to barriers to healthcare access during the COVID-19 pandemic, have accelerated the integration of the IoMT into medical practice (10). There is a growing pipeline of medicines and medical devices in the clinical phase, supported by the use of digital elements such as wearables, smart phones, and digital endpoints (11).

In terms of medicines development, the IoMT has the possibility to increase the robustness of clinical trials through enhanced data collection. For example, it can improve recruiting patients into trials, monitoring endpoints and patient responses, as well as generating real-world data outside of trials, for regulatory purposes. After their approval, it can also facilitate the monitoring of medical products. Nevertheless, the IoMT brings challenges, for example, in the validation of the broad range of digital use cases, of the data streams (vis-à-vis the conventional deliberate collection of data points), of new digital biomarkers and endpoints with their underlying devices, regulating data management, data governance and their changes/updates, as well as the integration of devices across care and clinical trials (10).

In the future, the IoMT will likely grow, and the EU is preparing by strengthening the interface between medical device and medicines regulation, by expanding its competencies in the areas of big data and artificial intelligence (AI), and continually supporting and learning from incoming use cases (12). Work is also in progress to develop the framework of interaction between medical devices and medicines assessment, to increase competencies and expertise gaps to regulate the IoMT.

#### Brain-computer interfaces for treating neurological disorders and enhancing health

A brain-computer interface (BCI) is a device that measures, processes and, in some cases, stimulates neuronal activity to generate a person’s response (13). Such devices are being explored for rehabilitation of motor impairments, as well as for augmenting human capacity either physically or cognitively. These interfaces can also control assisted living devices and artificial limbs that can provide sensory feedbacks to the nervous system. One example of an invasive BCI is an FDA-approved device capable of detecting epileptic seizures fingerprints in brain cortical electrical activity and delivering electrical brain stimulation (14). Several European clinics offer BCI-based neurofeedback training for the treatment of neurological disorders or for health enhancement purposes.

As medical devices, BCIs need to be assessed for safety and performance and require analytical and clinical validation (for the regulatory challenges associated with AI see Artificial intelligence). Depending on whether a surgical implantation of the device is needed, there may be specific safety concerns associated with this procedure; the same goes for the electrical stimulation of the brain tissues where there are risks for over-modulation and wearing-off effects that need to be carefully evaluated in clinical studies.

In medicines development, BCIs may be used as diagnostic tools, to derive endpoints, including disease progression, and to support the administration of personalized medicine doses. However, the extent to which these devices will be used in conjunction with medicines development is unclear, with limited information available in clinical trials databases. Whilst single-arm trials and sham controls are being used, these have their own scientific and ethical challenges. To identify and address the ethical issues like privacy, freedom/autonomy, personal identity, brain hacking, psychological wellbeing and safety of the patient, neuroethicists are collaborating with developers (15).

In the coming years, it is expected that the dividing line between medicine and device continuously becomes less clear and such technologies further develop in the direction of personalized treatments. The latest discoveries in the field of interoception, i.e., the sense of the internal (health) state of the body, together with the continuous advancements in neuroscience and AI technologies could expand the range of applications of BCIs to non-brain related conditions (16).

#### True personalized medicine

The European Commission’s Horizon 2020 Advisory Group defined personalized medicine as “a medical model using characterization of individuals” phenotypes and genotypes (e.g., molecular profiling, medical imaging, lifestyle data) for tailoring the right therapeutic strategy for the right person at the right time’ (17).

Currently, clinical development of personalized therapies is most commonly found for genetic disorders, such as cystic fibrosis, epilepsy and cancer, or in the context of cell therapies. In these fields, medicines are now being developed for specific sub-populations that share common features. These developments that challenge the traditional “one-size-fits-all” approach to medicines development, have been enabled by progress in the field of predictive biomarkers and companion diagnostics, driven by the integration of “omics approaches (in particular genomics, transcriptomics, proteomics, epigenomics) in clinical practice. They have also been enabled by phenotypic clinical and real-world data for identification of new potential biomarkers and pharmacological targets (18–20). This “omics toolkit” is enabling medicine platforms rather than specific products, supported by decentralized multiple product manufacturing (21, 22).

In the next 10 years, the forefront of medicine will likely advance further, beyond the personalized prescription of medicine, to the personalized manufacturing and more individualized dosing of medicines. Truly personalized medicines will optimize the choices and dosing of a medicine (likely produced individually for the patient in a local healthcare facility) and of treatment sequences for a given patient based on individual preferences based on individual effect predictions as well as individual biological features of the patient and his or her condition as well as effects observed in the clinical practice for the respective patient. This “truly personalized medicine” has the potential to help improve the effectiveness and safety of medicines provision and alleviate rising healthcare costs of more prevalent chronic diseases and an aging population (18).

There are several ongoing EU regulatory science research projects which strive to advance the field. Personalized Medicines Trial (PERMIT) is a H2020 funded project whose goal is to develop recommendations for robust and reproducible personalized medicine research (23). Another example is the International Consortium for Personalised Medicine (ICPerMed), which establishes a platform for sharing knowledge about personalized medicine research, its funding and implementation (24, 25).

Moving to truly personalized medicine requires new regulatory approaches for the oversight of decentralized manufacturing, individualized benefit-risk, innovative trial designs, novel biomarkers and digital endpoints use, amongst others. Its implementation within healthcare systems will require the development of new competences and of sustainable economic models that allow for these improved diagnostic, therapeutic and preventive approaches (18, 21, 22, 25, 26).

#### Plant-based production of edible vaccines and therapeutics

Plants are a prominent alternative system that can be engineered for the manufacturing of therapeutics such as small molecules and biologically active recombinant proteins, including edible vaccines. The first plant-produced therapeutic protein that obtained a regulatory approval for human use was taliglucerase alpha for the treatment of Gaucher’s disease, produced in carrot cell culture (27). However, the development of plant-based therapeutics seems to be falling behind other systems, in particular recombinant systems.

The relative novelty of plant-based production of vaccines and therapeutics leaves regulatory uncertainties which could in part limit their uptake. They also include factors influencing quality and impurities determination, including harvesting timing, medicine composition (such as the effects of non-human glycosylation and immunogenic sugars) and tolerance.

Another risk potentially limiting the use of plant expression systems is the accidental contamination of crops for human and animal consumption with transgenic plants. This risk is sought to be minimized with several approaches advanced in legislation (28). Additionally, the presence of non-human post-translational modifications, which is an issue for all non-human expression systems, can cause an adverse immune response (28). As a result of these barriers, the majority of plant-based products available in the market are either diagnostic, veterinary or classified as medical devices (28).

Despite this limited uptake, plant-based manufacturing of recombinant proteins could be done rapidly, cost-effectively, and with potentially higher scalability and flexibility compared with other expression systems, meeting the high demands observed during public health threats such as the Ebola virus outbreak (28). Furthermore, it has the potential to limit the need for complex purification. An additional advantage of plant-based proteins is that they do not produce endotoxins nor support the growth of viruses or prions (28).

One interesting area of plant-based production are edible vaccines produced from genetically modified plants. These deliver an antigen *via* the digestive tract mucosa and stimulate a mucosal immune response. The edible nature of plant-based vaccines means that they would reduce the need for medical personnel to administer (29). They may also lower rates of vaccine hesitancy, alleviating fear of needles and concerns over other production methods, such as seen with vaccines produced in HEK 293 cells. This could help address unmet medical need in pandemic situations.

Ethical concerns may arise from the interest of certain lobbyists of industries related to the production of plant-based goods (for example: the tobacco industry), which can support developments in the field of medicines.

In summary, plant-based production of therapeutics and vaccines has notable potential benefits but its uncertainties need to be reduced through gaining more experience and support.

#### Genetically-engineered phage therapy

Phage therapy has gained interest as a tool to treat infections resistant to antibiotics. They consist of viruses (bacteriophages) that attack the pathogenic bacteria but not human or other animal cells. Phage therapy can use off-the-shelf cocktails or be individualized for a given patient by identifying the bacteria causing the infection, testing for phage susceptibility and creating a therapy from one or more efficacious phages (30). Phages co-evolve with bacteria in the environment and have a very narrow spectrum antibacterial activity, therefore, the selection of phages against a patient’s bacterial isolate can require screening a phage bank. Phages can be naturally occurring or genetically engineered to improve their precision in targeting bacteria.

The Agency has seen a few examples of phage therapy over the years in ITF briefing meetings and in scientific advices for veterinary medicinal products, though none have applied for marketing authorization. In the EMA business pipeline, bacteriophage cocktail formulations and some personalized approaches are currently being developed, with no phase 3 trials and only a small number of phase 1/2 interventional trials, most of them having started recruitment in recent years. In fact, so far, no randomized control trial has shown the efficacy of this type of product.

The individualized nature of bacteriophage therapy makes assessing their safety, efficacy and quality potentially challenging, in particular because phage therapy may be tailor-made for each patient, contrary to the concept of a standardized medicinal product. There are also several knowledge gaps for phage therapy including how host immunity effects their efficacy and safety, how phages interact with eukaryotic cells, as well as phage distribution, accumulation, and persistence within the organism (31). General concerns exist around how phage therapy will select for resistant bacteria in patients and the environment, and the extent to which this resistance will be spread across species (32). There are also ethical issues as to whether phage therapy clinical trials can be blinded and should be conducted with comparators or placebo in case of resistant infections. The lack of robust scientific evidence of efficacy, the knowledge gaps and the limited experience in phage therapy means that currently, there are few regulatory guidelines relevant to phage therapy (33, 34). It is unclear in how far the manufacturing of phages can deviate from the manufacturing and quality standards used in viruses for vaccines, for example. Although a guideline at this time would be premature, EMA organized a workshop on the therapeutic potential of Phages in June 2015, where issues were discussed between different stakeholders.

Over the coming 5 years, phage therapy is not expected to apply for marketing authorization, but EMA expects to see more come to scientific advice or ITF. Given the potential public health importance of phage therapy to combat antimicrobial resistance, a growing pipeline over the coming 10 years is anticipated and should be supported.

#### Improving pandemic preparedness systems

The most serious pandemic for a century, COVID-19, has caused unprecedented global scientific, societal and policy responses. The pandemic preparedness systems in place before COVID-19 have been put to the test and there are now many initiatives to learn lessons to improve preparedness for future pandemics.

From an EMA perspective, several such lessons can already be drawn (2, 35). These include the need to maintain the EMA pandemic Task Force, ensure mechanisms for rapid scientific advice, promote and prepare for large, well designed clinical trials, invest in and optimize (real-world) data collection, strengthen inter-agencies and international collaboration, build upon necessary communication strategies and support coordinated EU-level evaluations of emergencies and interventions.

The EU is working to implement these lessons, and improve pandemic preparedness for the future, including extended mandates of EMA, according to Regulation (EU) 2022/123, which became applicable in 2 February 2022, and the European Centre for Disease Control (ECDC) in public health emergencies to include the monitoring and mitigating of shortages of medical products and devices (36); transferring to EMA the task of managing the “EU Expert Panels” for clinical evaluation of certain high-risk medical devices and *in vitro* diagnostics; requiring EMA to invest in and leverage real-world data to support crisis preparedness and response; enhanced ability to fast-track regulatory opinions and authorizations of pandemic medicines. EMA’s reinforced role was presented in a multistakeholder workshop held in April 2022 (37). This may pose challenge to existing “peacetime” regulatory processes and resources for assessing the safety, efficacy, and quality. However, this challenge will likely scale in proportion to the public health need of a future pandemic.

Beyond the expanded mandate of EMA and ECDC, a new EU authority has been established to further EU preparedness and response to serious cross-border health threats: the European Health Emergency Preparedness and Response Authority (HERA). HERA will enable the rapid availability, access and distribution of medical countermeasures. Public bodies like HERA and EMA also have a role in facilitating the pre-preparedness of vaccines for potential pandemics, funding or encouraging non-/clinical data to be gathered in advance for vaccines against pathogens with pandemic potential.

It seems likely that, at a global scale, better preparedness in the future to spot infections with pandemic potential in animals and humans will contribute to a faster response to prevent another global pandemic. Once an outbreak with pandemic potential has been spotted, an agile regulatory response and medicine R&D involving the newly established vaccine platform technologies with deeper production and logistics capacities, should bring medicines to market sooner. This is exemplified by the response to the monkeypox outbreak, which is closely monitored by the EMA. EMA remains in close contact with medicine developers to support R&D and provide regulatory advice (38). Whilst the ability to implement pandemic preparedness are often at the national level, global institutions demonstrated the need to maximize joint preparedness, joint prevention and joint response. In addition, global cooperation also plays an essential role for equitable access of medical countermeasures (39).

#### Genome editing for rare and common diseases

There are a number of promising genome editing tools being developed, including transcription activator-like effector nucleases (TALENs), zinc finger nucleases, clustered regularly interspaced short palindromic repeats – CRISPR-associated protein 9 (CRISPR-Cas9) editors and prime editors. These can be used to modulate, repair, replace or add genes to achieve a desired genotype with the potential to cure diseases. These tools are highly promising, but challenges remain such as caused by off-target editing, which can cause potential safety issues associated with the unintended cleavage and modification of other genes in the target cell or others, including with the risk of causing germline modifications. In addition, there is the need for improved modes of delivery. To this end, continual advances are being made to make genome editing more precise, more efficient and less expensive, and this trend is set to continue (40).

As of March 2022, there are no medicinal products authorized in the EU which employ genome editing. However, more than 60 companies are developing medicines based on these techniques, and several such products are now in clinical trials for a range of *ex vivo* and *in vivo* applications. The genome editing medicines currently under development have focused on gene reactivation or deletion, *ex vivo* rather than *in vivo*, for diseases such as sickle cell disease (40).

In the future, genome editing tools will have greater precision, enabling editing *in vivo* and for multiple edits. Additional features will also be incorporated including safety features such as self-inactivating genome-editing tools which limit the time that products are editing and reduce the potential for off-target effects. Reporter and “suicide” genes are also being incorporated to track the fate of treated cells. Another future possibility will be personalized edits applied to treat diseases for which there are hundreds of different disease-causing mutations, such as cystic fibrosis. Here, validated *in vitro* testing alone may have to underpin regulatory assessment of genome editing technologies that have already been well-characterized in previously approved medicines (41).

To enable this continued innovation in genome editing products, more will need to be understood about their immunogenicity, off-/on-target effects, dosing and their long-term clinical safety and efficacy. The ethical considerations of genome editing are also needing to be advanced, having mainly been used *ex vivo* on somatic cells from patients with a clear human disease phenotype. The possibility for germline genome editing and functional enhancement raises ethical and regulatory concerns that will need to be addressed with all stakeholders when it is appropriate, whether and how it could be conducted safely and what evidence would allow for an intergenerational benefit–risk evaluation (42).

#### Artificial intelligence

AI technologies are present and are being applied across all stages of a medicine’s lifecycle: from target identification, candidate optimization, analysis of clinical data in trials, to pharmacovigilance and clinical use optimization (43). It is set to have an increasingly large impact on medicines development, regulation and healthcare delivery. Overseeing the use of this AI challenges regulators throughout the lifecycle of a medicine, as it is increasingly being applied to, for example, target profile identification and validation, compound screening and lead identification, biomarker identification, digital clinical endpoints, dossier preparation for submission and processing of adverse events (44).

There are several hundred clinical trials ongoing that refer to “artificial intelligence,” with applications across therapeutic areas: to enhance or assist diagnosis, screening and image-based procedures, support medical decisions and clinical management, contribute to the establishment of new biomarkers or endpoints and as an integral part of medical devices, etc. (45).

EMA already encounters products utilizing AI, for example digital biomarkers, and this is likely to increase over the coming years as AI applications increase in effectiveness and impact. This range of applications brings with it regulatory challenges, including the transparency of the algorithms themselves, their meaning, and managing the evolving nature of these algorithms. In order to be able to assess and validate AI applications in the context of a medicinal product, a new regulatory framework will have to be developed, entailing new guidance and standards, data governance, variation procedures and enhanced regulatory capacity. This framework should be risk-based with respect to the benefit/risk, with regulators having the expertise and access to interrogate the underlying data and algorithms. It will also need to consider the governance of decision-making on the use of AI and its ethical implications.

#### Telemedicine and digital health transformation

The digitalization of healthcare is enabling its data-driven innovation, delivery and assessment. From a health systems perspective, digitalization can help integrate care through electronic health records, predict care demand and enable improved patient treatment. Key enablers of innovation in healthcare are platforms of data exchange and analyses, enabling, for example, predictive clinician decision support software and remote assessment in telemedicine appointments (46).

In medicines development, digital health will allow a range of innovations, from novel, patient-centered biomarkers, pragmatic trials, register-recruiting trials, recruitment of patients earlier in their disease course, more rapid recruitment and more efficient and granular clinical trial documentation. For example, wearables already provide for the continuous collection of patient data, assisting medicines development and treatment (47). Digitalization should also enable improved patient enrollment in clinical trials and in particular decentralized ones, as well as more observational research on medicines use (48).

Currently, digitalization is heterogenous across the EU and this is likely to remain the case due to the fragmentated nature of healthcare systems, culture, skills, governance and incentives (46). Nevertheless, the EU is laying out the governance and regulatory groundwork for digitalization with, for example, health data standards for data interoperability as well as the coming European Health Data Space (49).

From a regulatory perspective, this transformation is already posing challenges with the rise of wearables, AI and the need to better utilize data originating from the healthcare system, complementary to clinical trials. Increasingly, regulators will have to face strategies that propose to utilize this kind of evidence instead of traditional clinical trials.

In the next 10 years, advances in digital health, and strides made during the COVID-19 pandemic, should facilitate the greater update of telemedicine (50). Consumer expectations, and experience with robust digitalization in fields such as banking may further demand and thus supply. This should help to alleviate geographic inequities in healthcare access; however, the opposite effect, due to lack of digital literacy or affordability of the devices, is a risk. It has been reported that AI will also benefit from the digitalization of healthcare and allow for automated decision support for healthcare professionals in patient diagnosis, counsel and treatment (51). Finally, medicines regulators may be able to use real-world date to monitor medicines effectiveness in the real-world (52).

#### Climate emergency, health needs, and regulatory response

Climate change will have a negative impact on public health through, for example, heat-related diseases and functional impairment, changing infectious disease epidemiology, extreme weather events, forced migration and stress (53). This may aggravate the risk of pandemics and other public health emergencies. Tackling the climate emergency will require a global approach across sectors, including pharmaceuticals, which are a large source of environmentally damaging emissions such as greenhouse gasses of the healthcare system footprint (54). This may entail stricter environmental management of healthcare provision, including medicine development and use, minimizing traveling of patients and researchers through more telemedicine, remote trials, remote scientific meetings, the prudent use of excessive power consumption for big data and iterative, intensive computations (AI), the use of fewer consumables etc.

For medicines regulation, this may have a direct impact on the development and regulation of medicines for newly prevalent infectious and functional diseases. New guidelines, and deviations from them, for example in GMP, may become permissible for environmental reasons.

Governments globally are responding to climate change and the European Union has committed to reducing greenhouse gas emissions by 55% by 2030, compared to 1990 levels, and the European Green Deal sets the goal of making Europe the first climate-neutral continent by 2050 (55) further supported by the European Climate Law [Regulation (EU) 2021/1119]. This requires action also throughout the lifecycle of medicines to reduce resource use, emissions and levels of in the environment. The European Commission’s Pharmaceutical Strategy also contains actions to mitigate environmental damage caused pharmaceutical residues and the impact on antimicrobial resistance (56). These actions can be facilitated by e.g., improved environmental supply chain management and transparency, and environmental risk assessments which should be in line with the EU’s Strategic Approach to Pharmaceuticals in the Environment (57).

In addition, EMA’s activities are also being adapted to toward the goal of reduction of emissions with Committees operating in hybrid between remote and face-to-face mode, reducing therefore travel related emissions.

Over the next 10 years, substantial emissions reductions of medicines development, manufacturing and supply chains, and enhanced environmental risk assessments will be necessary. The extent of these, and role of medicines developers and regulators, remains unclear.

#### Antimicrobial resistance

Despite the global commitment to tackle antimicrobial resistance (AMR), it still represents one of the biggest threats to public health. Murray et al. (58) estimated a global 1.27 million deaths in 2019 directly caused by AMR, the largest share of the burden being associated by WHO’s priority pathogens. The Global action Plan on AMR by the WHO (59), the European AMR Action Plan (60) and the Food and Agriculture Organisation (FAO) Action Plan on AMR (61) address the issue as a One Health challenge, which means that it is equally important to combat the development of antimicrobial resistance in human health, in veterinary medicine and in the environment, since the three are interlinked.

There are several areas where action can be taken to combat AMR in both human and veterinary medicine. In addition to raising public awareness, infection prevention and control measures, including promoting hygiene measures and improving clinical practice to reduce infections, are tools against the development and spread of resistant infections. Besides this, the use of antimicrobials can be optimized with stewardship initiatives which rationalize prescription to minimize the emergence of resistance. Rapid diagnostic tests, delaying prescriptions or personalized dosing through therapeutic drug monitoring are useful approaches in this regard (62, 63). Surveillance systems collecting data on antimicrobial consumption and the development of resistance are also essential to guide policy and research (64). Action is also required to reduce antimicrobials in the environment, and the European Commission is working on strengthening environmental risk assessment requirements and ensuring safe disposal of medicines. To shepherd the multitude of initiatives combating AMR, a collaborative approach across nations and sectors has been established over the past years.

Due to the evolving nature of pathogens and the selection pressure exerted by antimicrobial medicines, the above-mentioned measures can only delay the occurrence of resistance and alternative treatments for infections are still highly needed. The current clinical development pipeline remains to be dominated by traditional antibiotics, mainly derivatives of known classes, as elucidated by WHO’s 2020 report on the clinical pipeline of AMR relevant products (65), in which it is predicted that 8 new such antibiotics may receive approval in the next 5 years (65).

The developments in new antibacterial agents are led by SMEs. To address the issue, funding gaps in the initial phases of development will need to be overcome and, most importantly, the market failure which has led “big pharma” to largely leave this field, will need to be tackled with the creation of new “pull incentives,” which decouple profit from sales.

Over the next 10 years, increased support to research and development of novel antimicrobials or alternative therapeutics is likely: implementing appropriate business models (pull incentives), push incentives and regulatory incentives (66). These regulatory frameworks and guidances should provide for the development of vaccines, phage therapy, novel biologically active molecules such as peptides or enzymes, non-specific immunomodulators, gene editing technologies and photo switchable antibiotics (67–69). The development of products used as adjunct therapies should also be encouraged (70, 71).

Many of these alternatives to conventional antibiotics pose regulatory challenges: their new mechanisms of action; their biological/chemical complexity; the individual and personalized nature; clinical trial design, especially the recruitment of patients with bacterial infections with resistant strains; the use of appropriate comparators and the difficulties in demonstrating efficacy of adjunctive therapies (67, 68, 72, 73). Although funding and other incentives may increase the number of new developments, surveillance systems and stewardship approaches will have to continuously adapt to guarantee that the therapeutic solutions are sustainable in the long-term.

#### Healthy aging

Thanks in part to medical progress and better living conditions, global life expectancy has increased over the past decades, but this trend has not been accompanied by a proportional gain in years spent in good health. Therefore, a significant challenge to society will be handling the social and economic burden of an increasing elderly population at risk for multiple chronic age-related diseases, more susceptibility to communicable diseases and greater economic and functional dependency. The strategy adopted by a growing number of institutions consists in preventing, or delaying, the onset of age-related poor health by promoting healthy aging (74).

A variety of molecular pathways have been linked to aging. Further research is needed to elucidate these processes and clarify the causality of their interactions (75). The biological complexity of the natural aging process does not fall within the commonly understood definition of disease. It is not currently conceivable to grant an indication for “aging,” and the regulators rather focus on measurable symptoms such as mobility. This complexity of the ageing process also makes targeting healthy aging challenging for novel therapeutics (74). Similarly, defining the type of overall benefit that a treatment should aim for, its pharmacodynamics and biomarkers, and how this can be assessed may be uncertain. Currently, the outcomes of animal model experiments and clinical trials are presented in terms of function or life enhancements, life extension and prevention of age-related diseases (76).

“Anti-aging” therapies therefore pose several challenges for medicines development and for the EMA.

The above-mentioned knowledge gaps make it challenging to assess dosing and efficacy, corroborated by the lack of interim endpoints and pharmacodynamic parameters, in particular for CNS-directed therapies. Anti-aging approaches likely require large and long clinical trials (74). The prevalence of comorbidities in the targeted elderly population increases heterogeneity and is a challenge to safety and efficacy assessment. The vulnerability of such a population may also raise ethical concerns for clinical trials, especially in terms of the level of expected risks and burden that may be acceptable in view of the absence of a standard of care (76). The specific regulatory requirements for conducting trials in a population of older adults are reflected in the EMA, which published a Q&A document on the ICH topic E7 “Studies in Support of Special Populations: Geriatrics” (77) and more recently rediscussed in the FDA’s workshop “Roadmap to 2030 for New Drug Evaluation in Older Adults” (78).

One example of developments in the healthy-aging field are “senolytics,” which induce the death of senescent cells. Recently, such a senolytic entered first-in-human trials with preliminary, but seemingly promising results (79). Other strategies include calorie restriction, NAD + supplements, mTor modulators, exercise and other interventions (76).

While these knowledge and regulatory classification gaps are being closed, the repurposing of existing medicines to aid healthy aging presents several advantages over *de novo* drug discovery, as much of the data and experience for regulatory purposes already exists. Other promising, perhaps more potent approaches should be supported in consideration of the long time needed for their development, such as gene therapy, genome editing, plasma-derived therapy, fecal microbiota transplantation and stem cell rejuvenation (80).

In addition, numerous initiatives have been launched at European level by the European Innovation Partnership on Active and Healthy Aging, aimed at strengthening research and innovation, and by the AGE platform, focused on policy areas that impact older and retired people (81). Additionally, the United Nations has proclaimed 2021–2030 the Decade of Healthy Aging, to raise awareness and intensify global collaboration in this area. These projects include, for example, strategies to extend a healthy life-span, such as digital solutions to support wellbeing in people with chronic disorders by promoting an active and healthy living (e.g., *via* virtual coaches and strategies for social integration) and also initiatives to establish a system of integrated, personalized care and detection of early signs of disease, often resorting to innovative technologies, such as big data, AI and robotics, among others.

Over the next 10 years, given the high burden of age-related diseases to society, healthy aging approaches are expected to increase in number. Although examples of successful repurposing could be seen in a nearer future, more innovative approaches are more distant on the horizon.

### Joint Research Centre health

The topics below were derived from the 156 signals relating to health resulting from the DG JRC horizon scanning exercise (7).

#### Epigenetic treatments

Epigenetics involves the interaction of DNA-related proteins with DNA or RNA; for example, DNA methylation, histone modification and non-coding RNA. It regulates gene expression and can cause, and affect, disease pathology. A prominent example are H3 K27-mutated diffuse midline gliomas (82). This mutation alters a site critical to post-translational modification in the histone which impacts the DNA methylation and consequently gene expression, driving gliomagenesis (83, 84). Epigenetic information is inherited and can have environmental causes, including infections. For example, Mycobacterium tuberculosis (MTB) epigenetically modifies macrophages, suppressing their IL-12B gene and enhancing MTB survival (85). As a result, epigenetics can be used in diagnostics or treatment for diseases, such as certain cancers that are causally associated with epigenetic changes.

Epigenetics is a field where we are still expecting the new knowledge gained over the past decades to systematically translate into innovative diagnostics and treatments. Strategies such as inhibition of DNA methylation and histone deacetylation are examples of authorized non-specific epigenetic modifiers which found their way to the clinic and are being further explored in clinical trials (86). However, therapies that work through targeted epigenetic modifications are novel. Only a handful of these are in clinical development and are using the technologies of genome editing, such as zinc-finger proteins, transcriptional activator-like effectors and CRISPR (87). Similar modifications can be made to RNA, sometimes called the epitranscriptome, although corresponding medicines are not in the clinical phase yet (88).

Epigenetic treatments have a large potential but overall the science underpinning them is still thin in most diseases, and research is largely in the exploratory phase of mapping the epigenome, understanding mechanisms and disease associations, and so regulating such treatments could be challenging. The complex interplay of epigenetic factors that drive gene expression makes it difficult to select appropriate targets. Unforeseen effects can be caused by modifying a member of a bigger complex of proteins, and this may be difficult to recapitulate *in vitro*. Off-target effects are also a known concern. Screening assays must also consider the structure of the chromatin. Furthermore, there is uncertainty around the duration of therapeutic effects due to passive demethylation and deacetylation (89).

Over the next 10 years it seems likely that the development of targeted epigenetic therapies will increase, mainly in the field of oncology, although this will remain limited to a few products.

#### Computational ‘omics profiling

Molecular characterization technologies are evolving at a fast pace, enabling the extraction of multi-omics data from different types of biological samples. In parallel, there is a growing number of computational tools that facilitate the analysis and integration of these datasets, and this will provide more comprehensive information about human and animal physiology and diseases (90).

Currently, ‘omics analysis is mainly applied in the research context and is narrowed down to the analysis of selected panels of biomarkers in the clinical setting (91). However, these technologies will acquire an increasing role in personalized clinical decision-making, particularly in oncology: addressing variability among patients, intra-tumor heterogeneity, drug resistance mechanisms and influence of the immune system (92).

Computational technologies are expected to play a significant role in unraveling the multiple determinants underlying diseases. The identification of novel biomarkers, likely in the form of “signatures.” will promote a shift toward histology-independent medicines, combinatorial therapeutic approaches, and preventative treatments. Omics profiling could become determinant not only for the diagnosis and monitoring of diseases, but also for medicine development, guiding the choice of preclinical models and the recruitment of patients in clinical trials (90).

These new computational tools may require validation and qualification (93), which can be challenging due to the lack of gold standards, the complexity of the software and the multidisciplinary expertise required (94). Furthermore, many ‘omics computational tools fall under the *in vitro* diagnostic medical device regulation, where it may be difficult to map the requirements and the corresponding characteristics of the computational tool. For example, clinical performance will require a specific definition of the purpose of the approach, which can also potentially pose problems when the intended use is multi-faceted (95). In this case, analytical and clinical performance is difficult to separate, although these are the responsibility of two different entities, namely Notified Bodies and National Competent Authorities/EMA, respectively.

The development of computational ‘omics profiling will most likely continue to expand over the next decade, aided by developments in machine learning and quantitative systems pharmacology. It will bring new understanding of disease mechanisms, especially cancer biology, which could be fundamental for the discovery of innovative medicines. The implementation of future ‘omics will require additional expertise, including diverse teams of technicians (IT, AI, software, etc.) and clinicians within medicines regulators and in routine clinical practice where multiple disciplines are required for generating, analysing and presenting data (96).

#### Circular and long non-coding ribonucleic acid

Advances in transcriptomic analysis are enabling the functional characterization of different types of RNAs that act as important biological interactors. Various circular RNAs (circRNAs) and long non-coding RNAs (lncRNAs) are gaining attention due to their involvement in the regulation of physiological and pathological processes. LncRNAs constitute opportunities to expand on the targets of small interfering RNA (siRNA) or CRISPR-Cas9 based technologies or explore new approaches based on circRNAs or natural antisense transcripts to achieve innovative therapeutics (97).

Circular RNAs are transcripts closed in a loop after back-splicing events. Their biological function is currently being investigated but emerging data show that only a minority are translated into peptides. Among the functionally characterized circRNAs, several interact with other non-RNA biomolecules and regulate transcription, translation and miRNA transport (98).

LncRNAs are transcripts containing more than 200 nucleotides that are not translated into proteins. Contrary to the definition, a subset of lncRNAs can be translated into short polypeptides, but the majority interact with other biomolecules and regulate replication, transcription, post-transcriptional modifications, epigenetic modifications and DNA repair (99, 100).

The expression of circRNAs and lncRNAs appears to be tissue specific and dependent on the developmental stage of the tissue or organism. Several public databases contain the annotation and functional description of these transcripts, many of which have also been correlated with pathological conditions. The validation of analytical tools that avoid mis-annotations in transcriptomic studies is one of the challenges to overcome to facilitate further developments. The usual concept of open reading frame (ORF), which is a sequence limited by start and stop codons of a length corresponding to about 100 amino acids in eukaryotic cells, is not directly applicable (101).

There is growing evidence that these RNAs might play a role in cancer, neurodegenerative diseases, aging, autoimmune diseases, immune responses, and drug resistance mechanisms (102). Currently there are about a hundred clinical studies listed that investigate the role of circRNAs and lncRNAs as potential biomarkers, while the development of therapeutic strategies targeting these transcripts is still in the preclinical phase. Besides, the identification of appropriate non-clinical models that recapitulate the complex interactions in the human also remains challenging (103).

There are currently no examples of circRNA- or lncRNA-based therapeutics being developed at a clinical stage, and therefore it is not very likely that they will be available in the next 10 years (97). As with other RNA-based strategies, problems with specificity (on-targets in cells other than intended and off-targets), delivery (instability of naked RNAs, inefficient intracellular uptake) and tolerability (adverse immune effects) slow the translation into the clinic (97).

#### Fecal microbiome transplantation for clostridiodes difficile

Clostridioides (formerly Clostridium) difficile (c. difficile) infection (CDI) is an intestinal infection with a mortality of up to 34% for patients in intensive care units (104). The first line treatment are antibiotics, however, their use disrupts the intestinal flora and does not eliminate the C. difficile spores, which renders recurrence (rCDI) frequent, affecting roughly 25% of patients (105).

Several clinical trials are ongoing for FMT with indication for CDI, mostly in phase 2, some of which are exploring new formulations or routes of administration. FMT is also currently being investigated for treating other indications, including GI tract diseases, other infections, metabolic syndrome, graft vs. host disease and cancer.

At the moment, there is a lack of regulatory clarity surrounding FMT. In Europe, there is no standardized policy in place, and it is the responsibility of each Member State to decide on the applicable framework (106). This leads to heterogeneous classification of the treatment and subsequent approaches in the EU (107).

Furthermore, FMT also poses challenges regarding safety, quality, and efficacy. There seems to be a consensus around the notion that donor selection and screening must be very strict, in order to control for infectious diseases and even GI, metabolic and neurological disorders. Although these measures increase the safety of the procedure, there is no clear understanding of what a healthy microbiome is, or if it even can be defined (108–110). Moreover, it is important to establish long-term safety monitoring systems (107, 111).

From a quality perspective, FMT is very complex, and it appears impossible to ensure consistency from batch to batch, which compromises conventional quality control procedures (110). The EU Innovation network (EU IN) of medicine regulators’ report on FMT contains a more complete discussion of the regulatory challenges and of additional topics, together with recommendations to address them (112).

Over the next 10 years, assuming the mode of action is more robustly scientifically characterized and understood, with the microbiome elements responsible for the suggested health benefits identified, FMT could be developed successfully and adopted more widely and beyond CDI.

#### Cyber-biosecurity future technology

Cyber-biosecurity is a relatively new discipline that integrates cybersecurity, cyber-physical security, biosecurity and biosafety (113). The field of life (bio)sciences is increasingly dependent on cyber-physical domains. For example, new innovations are increasingly derived from bioinformatics, open access databases, artificial intelligence (AI) and automated equipment (114). In medicines development, cloud-based computing and data are increasingly used to develop therapies and to produce them. All the above make life sciences and current biotechnology highly vulnerable to threats such as unwanted (cyber) surveillance and harmful activities.

Apart from the risk posed to the security of personal medical data, and intellectual property, vulnerabilities in computers and networks can enable disruption, permanent destruction and malicious manipulation of biologic data, agents, materials of developers or databases of regulators, which can have devastating consequences on the environment, human and animal health (114, 115). Additionally, such events can undermine public trust in health and research institutions (116).

As medicines and devices are increasingly integrated, this also poses risks in a cybersecurity perspective. FDA offers guidance on pre-submission as part of the device safety and post-marketing requirements and educational resources to support developers of medical devices to correctly address cybersecurity risks (117). The latter includes tools to support threat modeling and communication strategies to patients. Regulatory requirements include, for example, aspects relating to security (authorization, confidentiality, integrity, and availability of data) and transparency and risk management.

This topic poses several challenges, including an inadequate awareness of the seriousness of the threats posed by cyber-bioattacks in the field of life (bio)sciences, a lack of expertise and relevant legislation, and challenges associated with the development of risk assessment and mitigation plans (115).

Currently, in the EU, regulatory guidelines or risk assessments protocols are not available. However due to the high risk associated with cyber-biosecurity and the ongoing shift toward “smart labs,” guidelines and recommendations are needed to ensure preparedness and harmonization. For example, additional regulatory requirements, such as new cyberbiosecurity rules in Good Laboratory Practice (GLP), Good Manufacturing Practice (GMP), and Good Clinical Practice (GCP) may be needed. In addition, relevant risk assessments and physical batch release testing, as well as digital auditing, may be beneficial for detecting for manipulations at a sub-batch scale.

The occurrence of cyberattacks has increased in recent years and cyber-biosecurity measures will have to be strengthened by all health domain stakeholders in the next years (118).

#### Disinformation and infodemic

Disinformation is “verifiably false or misleading information created, presented and disseminated for economic gain or to intentionally deceive the public” (119). It differs from misinformation in that the latter is not spread deliberately. Disinformation can lead to harm on an individual level, through misinformed actions, and at a societal level through damaged trust of institutions such as public health bodies and their recommendations. Disinformation has recently grown as a problem due to the rise of social media which has enabled disinformation to reach large audiences very rapidly and polarize information sources and debate, reducing exposure to counterviewpoints. Combatting disinformation is therefore a growing priority for public health authorities.

An infodemic, on the other hand, is defined as “too much information including false or misleading information in digital and physical environments during a disease outbreak” (120), as was observed during the COVID-19 pandemic. It can also lead to mistrust or confusion, undermining the public health response.

Public organizations such as the European Commission are undertaking initiatives to tackle disinformation (119, 121). EMA has been combatting disinformation, misinformation and the COVID19 infodemic by identifying it through social listening, “pre-bunking” false narratives before they are disseminated, and proactively communicating on aspects concerning citizens. In addition, enhanced transparency of EMA’s decision-making and stakeholder engagement to understand concerns have been used through, for example, public stakeholder meetings. In parallel EMA has been trying to simplify and amplify its communication *via* social media and through press briefings.

Over the next 10 years, it will be important to measure the effects of dis- and misinformation and on the uptake of vaccines, medicines and health outcomes, and these effects are certainly large. It will also be important to consider the effects of dis- and misinformation on clinical trial recruitment and representativeness, medicine adherence, as well as safety through misuse or substitution with of alternative medicines. This can inform the extent to which EMA should invest in evidence-informed countermeasures in the future. There is no simple solution but a need for a range of evidence-informed measures that learn from other fields which have been tackling disinformation, particularly climate change, and for collaborating with other agencies and public health institutions.

### Joint Research Centre BioChem

The topics below were derived from the 144 signals relating to biochemistry resulting from the DG JRC horizon scanning exercise. (7).

#### Personalized immunotherapy for “cold tumors”

“Cold” or “non-inflamed” tumors exhibit low or no response to immunotherapy due to lack of T-cell infiltration. They are characterized by a limited number of mutations, and subsequently limited neoantigen expression, which makes them resistant to immunotherapies. They are often found in breast, ovarian, pancreatic cancers and glioblastoma.

Therapies aiming at improving the immunogenicity of tumors include neoepitopes and neoantigen cancer immunotherapies (including the so-called “cancer vaccines”). Neoepitopes are novel epitopes expressed by tumor cells from patient-specific mutations. For example, “individualized therapeutic vaccinations” (122) are based on immunopeptidome and transcriptome analyses of each patients’ tumor. Predicted T-cell reactivity in each patient is used to guide the selection of peptide(s) or the mRNA sequence(s) to be included in the immunotherapy (123). So far, no clinically relevant response (tumor shrinkage) was reported in the treatment with cold tumors immunotherapies, however, the results to date show that neoepitope-based immunotherapies can generate tumor-infiltrating T-cell response in “cold” tumors, which shows the potential of this approach.

The current clinical pipeline, as searched in ClinicalTrials.Gov using the terms “neoantigen” and “vaccine” OR “immunotherapy” as intervention in July 2022, comprises around one hundred clinical trials investigating personalized neoepitope-based immunotherapies. Most of the trials are phase 1 only (92), 38 are labeled as phase II, and so far there is only one planned phase 3 trial listed. A search for“neoepitope” and “vaccine” or “immunotherapy” as intervention listed only 15 clinical trials, all phase 1/2.

Neoepitope cancer immunotherapies have several limitations which include high costs, issues with determining the optimal dosing strategy, measuring immunological changes predictive of cancer response, delivery including formulation, adjuvants and delivery vehicles (124). Furthermore, the manufacturing of such therapies must respond rapidly to demand, i.e., within days or weeks, in order to be used as neoadjuvant therapy. Difficulties of personalized/individualized manufacturing and batch-release also need to be considered.

From a regulatory perspective, the pathway for assessing these personalized products is unclear (124). In the EU, Cancer immunotherapies based on mRNA sequences for neoepitope expression are regulated as gene therapy medicinal products. Several aspects in the assessment of the quality, safety and efficacy have to be assessed and considered, including possible interactions between different administered or expressed peptides, approval of an individualized active substance (and possibly multiple such substances) as single medicinal product and defining the targeted patient populations, documenting safety and efficacy in pre-clinical and clinical development, the design of neoepitope immunotherapies based only on algorithms and AI (“*in silico*”).

In the future, the limitations will likely be overcome and the design, manufacturing and control of individualized neoepitope immunotherapies against cold tumors may use “a precisely defined, locked, verified (in the context of software systems), and repeatable process” (125). However, the regulatory challenges for their assessment and individualization require further conceptual work. From a public health perspective is it also relevant to support exploring how such immunotherapies might be used to “vaccinate” healthy populations against antigens from mutations which are prevalent and carcinogenic, including in cold tumors.

#### CRISPR-CAS9 application against human immunodeficiency virus

Human immunodeficiency virus affects millions of people worldwide; even though effective antiretroviral therapies are available to prevent and treat Acquired Immune Deficiency Syndrome (AIDS), patients are still at risk for non-AIDS-related morbidity and mortality. Non-adherence is partly responsible for this and the most common form of treatment failure (126).

Human immunodeficiency virus integrates into the genome of the host’s immune cells, and can lie dormant out of reach of antivirals. Genome editing offers the potential to address HIV in this situation. This approach has already shown to be promising using the genome editors zinc finger nucleases or TALENs in pre-clinical and early clinical trials, but now CRISPR is being tested for its low cost, adaptability and accuracy. By using RNA guides to target HIV-specific DNA, or cellular receptors that enable HIV entry, CRISPR cuts the host DNA to modify its expression. This can be used to enable latent reservoir cells to express HIV, so that it can be recognized and destroyed by the immune system (“shock and kill”), or to directly edit and delete the viral genes (127, 128).

The main challenge of CRISPR approaches are their on-/off- target effects which are difficult to characterize and therefore are challenges to the assessment of safety and quality. CRISPR-Cas9 also introduces mutations around the cleavage sites *via* the non-homologous end joining (NHEJ) DNA repair mechanism. These edits can lead to viruses that are resistant to editing with that CRISPR-Cas9 (127). The delivery of CRISPR-Cas9 is also challenging. The current efficacy rates of these gene therapies do not fully eradicate HIV sequences in all infected cells (129). Since HIV evolves rapidly, CRISPR would have to keep pace with its evolving target.

CRISPR-Cas9 is an approach likely to reach therapeutic clinical trials soon, with several mechanisms of action. There is an opportunity of combining this strategy with anti-retroviral agents to achieve higher potency (129). This high public health potential, along with its risks and uncertainties, should warrant supportive regulatory interactions with developers at an early stage.

#### Engineered living materials

Engineered living materials (ELMs) consist of viable cells enclosed in a matrix or scaffold that are used as advanced materials, similar to living organisms, for different applications in and beyond the health domain (130). In terms of medicine development, many are likely to be classified as tissue engineered products (131).

A growing number of such materials are currently being developed, making use of prokaryotic or eukaryotic cells, some of which have been genetically modified. These living materials can be assembled by cells producing their own support matrix, or *via* the addition of external substances, often natural or synthetic polymers, using technologies such as 3D printing and electrospinning (132).

In this way it is possible to obtain products with tailored properties, including the secretion of (therapeutic) molecules, the expression of molecules that respond to stimuli such as light, tension or magnetic fields and the customization of extracellular matrix properties for performance enhancement (133). This allows the creation of an advanced type of material capable of adapting to environmental cues, changing between different states and even self-heal (130). In the healthcare sector, ELMs can serve as preclinical models for *in vitro* testing or as drug production factories, with potential advantages in terms of environmental sustainability. Moreover, ELMs can be used as advanced medicinal products for tissue regeneration or sustained drug delivery; they could be personalized, incorporated into medical devices (possibly forming combined ATMPs) and also employed for diagnostic purposes (134). In the future, the field could evolve toward the creation of functional units or whole human organs for transplantation, but many challenges and knowledge gaps must be addressed to reach that stage (133).

Several regulatory challenges are associated with these products. Firstly, their classification as a (ATMP) medicine, device and/or blood cell and tissue product can be complicated. Attention will be required for the quality assessment and evaluation of the pharmacological properties of those materials intended for therapeutic application, particularly for applications where a “consortium” of microorganisms is used. The storage and preservation of the viability of the materials over time also need to be evaluated. Generating clinical evidence could be difficult due to the surgical nature of some ELMs, with difficult choices of control interventions. The living, changing nature and the irreversibility of some applications also challenges the regulatory assessment of their long-term safety and efficacy. Finally, careful environmental assessment will be required for GMO-based materials. All these aspects will require regulatory expertise in biomaterials.

#### Organelle-specific autophagy

Organelle-specific autophagy is an important cellular quality control mechanism that ensures self-preservation when organelles start to function abnormally, by degrading and recycling their components. The failure to control the dysfunction of organelles and the consequent accumulation of aberrant proteins is observed with different diseases such as inflammatory disorders and cancer. Thus, autophagy-based therapies may provide new therapeutic opportunities (135).

One such mechanism is reticulophagy, which is the autophagy of endoplasmic reticulum subdomains with faulty proteins and lipids and is linked to infectious and neurodegenerative diseases. For example, the gene RETREG1 (reticulophagy regulator 1) encodes a protein which works as a receptor (also known as FAM134B) that mediates remodeling and scission of ER sheets.

The interplay of the different organelle-specific autophagy mechanisms, which can be triggered by the same stimuli, by organelle cross-talk or even common overlapping pathways, increases the complexity of these mechanisms and the challenge of finding adequate and specific targets (135). For example, current cancer autophagy therapies aim to target the mammalian target of rapamycin (mTor), a protein that has an inhibitory effect in the process, and which is also involved in other cellular processes (136).

Currently, the use of autophagy in medicines development is focused on cancer, roughly 70% of clinical trials in which autophagy as a potential drug target has been investigated were dedicated to cancer, usually in combination with conventional therapies (136). Lysosomal inhibitors (for example, chloroquine and hydroxychloroquine) can be found in the pipeline at a later stage of development (137). Other therapeutic indications for autophagy under study include obesity, diabetes, cardiology, neurobiology and immunology, however the developments are mostly at an early stage (136).

One of the challenges is the so-called “double-edged sword” of autophagy-reducing therapies against cancer, which means that depending on the stage of a tumor, autophagy can suppress or support its growth and development (138). Besides the complicated efficacy assessment, there are also some safety concerns. Dosing and duration of treatment should aim to minimize overactivation of autophagy beyond clinical benefit in case of inducing agents, which could negatively impact the progression of diseases, especially in the cases of neurodegenerative diseases (139). On the other hand, excessive inhibition of autophagy could have a similar effect since it could result in aggravated inflammation. The lack of specificity of the treatments being developed could also have long term impact (136).

Given the complexity of the autophagy pathways, the lack of specificity of the therapies under development and the current knowledge gaps that remain, it is difficult to predict when autophagy-based therapies (even repurposed) will reach market approval, especially for cancer indications.

#### Proteolysis-targeting chimeras

Proteolysis-targeting chimeras, PROTACS, target and destroy proteins *via* the cell’s natural destruction pathway: ubiquitin proteasome system. PROTACs are made up of a target protein ligand and a linker to an E3 ligand, which enables the ubiquitin-proteasome system to degrade the target protein. They have the ability to target non-functional binding sites of proteins, with a high selectivity and therefore hold promise for previously “undruggable” targets of inhibitors, or for inhibitor candidates which failed due to non-specificity. In oncology, PROTACS are being investigated to target nuclear hormone receptors, epigenetic proteins, kinases and RNA itself (140, 141). They are also being investigated for skin disorders and neurodegenerative diseases, for example, targeting misfolded proteins (142).

Whilst PROTACS have been in development since 2001, their initial limitations in function and delivery have only recently been overcome. Their delivery options include administration as small molecules, antibody-drug conjugates or as RNA/DNA delivered *via* viral or nano vectors. They are now moving into clinical development, phase 1/2, mainly for oncology products (targeting, e.g., kinase inhibitors, epidermal growth factor receptor and androgen receptor).

There are some remaining scientific questions to address including the best delivery method for PROTACs, how to select the target and defining E3 ligase characteristics. Nevertheless, it is seems likely that PROTAC products could be submitted for Marketing authorization in the next 10 years (142).

#### Trained innate immunity

The immune system is traditionally divided in two: the rapid, non-specific innate immunity, which includes processes such as cytokine production and phagocytosis; and the slow, pathogen-specific, adaptive immunity, which is based on the targeted actions of dendritic cells, T- and B-cells (143). Several studies show that innate immune cells, after sensing pathogens or damage-associated molecular patterns, undergo an epigenetic and metabolic reprograming that alters their response to other stimuli, leading to a short-term “memory,” which can be trained (144). This non-specific adaptation has been defined as trained immunity and can also occur in non-immune cells. Stromal and epidermal stem cells, for example, demonstrated higher responsiveness to tissue damage after a first injury event (145). The Bacille Calmette-Guérin vaccine is an example of an approved medicine for the treatment of bladder cancer, whose mode of action consists of triggering trained innate immunity.

Targeting specific cellular pathways associated with trained immunity could allow an improved first line response against pathogens or tumor cells, or even the downregulation of the immune response in the context of inflammatory or autoimmune diseases. Therefore, trained immunity modulators could be helpful in addressing or preventing multiple pathological conditions. Furthermore, trained innate immunity is also thought to be associated with the aging process (143).

One of the challenges ahead for these treatment modalities is the identification of a correlation between the dosage of trainers and intensity and duration of immunological response. The mechanisms supporting the duration of these responses have not yet been elucidated and might involve other cell types such as bone marrow progenitors and tissue-resident cells. Myeloid cells, in fact, have a half-life of 5–7 days, while trained immunity can persist over months or years (146). Given the knowledge gaps around the pathways and cell populations involved, it might be challenging to find appropriate targets and to guarantee specificity to certain cell types. Finally, there is the potential risk of adverse inflammatory responses or other events caused by unspecific/unwanted changes in metabolism and epigenetics.

Further research will help clarify these aspects and allow the identification of endpoints and preclinical models to improve the translatability of novel therapies that rely on direct epigenetic or metabolic reprograming rather than the unspecific triggering of innate immunity. It is expected that a preclinical pipeline will follow this trend in the next years (147).

#### Tumor-derived extracellular vesicles

Recently, extracellular vesicles were found to play a relevant role in intercellular communication. There are several types of extracellular vesicles (EV) differing in size and origin, namely exosomes, microvesicles, apoptotic bodies and prostasomes. These can contain proteins, nucleic acid or lipids and originate from inward budding of the endosomal membrane and secretion from multivesicular bodies that fuse with the plasma membrane (148).

In cancer, they have been reported to fasten tumor progression through cargo which modulates the cancer microenvironment. Eliminating tumor-derived EVs from circulation seems to be possible using specific antibodies for cancer-associated surface antigens (148). Future cancer therapies could inhibit their release and absorption or promote their elimination. However, it is challenging to guarantee specificity of the inhibition strategies, which can interfere with pathways of normal cells. To rectify this, biogenesis and uptake mechanisms must be better understood.

In addition to being used as targets, tumor derived EVs could potentially be used as delivery systems for cancer immunotherapies. The major challenge to this application is the low loading frequency, especially in case of molecules that do not permeate a lipid membrane, such as nucleic acids (148).

Circulating tumor derived EVs and their cargo could also become useful biomarkers, derived from liquid biopsies, as they could be associated with disease status even in early stages (148). Currently, several ongoing clinical trials are studying EVs as diagnostic, prognostic, or predictive tools (149, 150).

It seems likely that the use of tumor derived EVs as biomarkers will continue to grow in the upcoming years. For EV based therapy, however, it seems there are still some fundamental questions which need to be answered, along with improvement to methods for isolating, purifying and storing EVs and analysing their contents in a high-throughput and time-efficient manner (148).

## Discussion

Many of the topics and innovations in this report are arising from advances in biomedicine and enabling technologies, and increasingly, from areas far beyond it. These bring with them an array of new opportunities and challenges. Horizon scanning, as conducted above, is a useful tool to systematically identify those that challenge current regulatory science methods for the assessment of medicines and which hold the most promise for patients.

Whilst there are benefits to the methods used to find and assess these signals, there were also limitations. These include methodologies which were not targeted toward medicines-related horizon scanning signals. This limited the relevance of the signals produced. However, in using signals from an un-biased “bottom-up” method, in addition to the “top down” approach, we hope this limitation was minimized and also that it identified signals that may not have been captured with a more targeted analysis. A second limitation was the use of semi-subjective filtration criteria to select the signals, as these criteria were qualitative and based on judgments that may be prone to researcher bias. Thirdly, the narrow assessment of each signal limited the insights provided but was necessary from a resource perspective and designed so that these assessments could help identify signals for future, deeper assessments. Finally, a regulatory perspective on these signals, whilst useful, is not sufficient to get a holistic view on their future utility to patients.

In this report, the horizon scanning signals detected by the WHO and the JRC exercises were assessed for their current status and potential impact over the next 10 years on the EMA. The broad diversity of the signals demonstrates the multidisciplinary and international nature of change and innovation. These will be used to future-proof EMA’s activities by looking into the most impactful signals in a greater depth and recommending how to prepare and reduce regulatory bottlenecks. For example, areas where there may be missing guidance which could create uncertainty and a barrier for medicines developers.

One such trend within medicines development is the line between medicines and medical devices becoming increasingly blurred by technologies such as brain computer interfaces engineered living materials and personalized therapies. The EU is making progress to align both regulations, but questions around the applicability of requirements in certain situations remain, along with gaps in latest expertise amongst regulators.

The COVID-19 pandemic has influenced some of the topics and innovations discussed here, especially in the field of digital health, where it has accelerated the uptake of telemedicine. The level of digitalization is currently variable across the EU, but governance and regulatory groundwork is being laid in this area. Digital health tools will continue to evolve in the coming years and pose challenges such as the (semi-)automation of diagnosis and prescribing. Digitalization also gives opportunities such as the possibility of enhancing the monitoring of medicines’ effectiveness in the real world based on data from healthcare processes, which may require revisions to the regulatory system and scientific processes.

The other topics which arose due to the pandemic were concerns associated with infodemic and cybersecurity. These are currently being tackled with renewed focus in the EU. One silver lining of the pandemic are efforts to improve pandemic preparedness. In this context, the mandates of the EMA (and other EU bodies such as ENISA, the EU Agency for cybersecurity) were extended, and a new institution, HERA, has been created.

In addition, the traditional concept of a medicinal product is being stretched by the personalization and individualization offered by forthcoming platform technologies. Several examples, such as RNA-based technologies, genome editing, epigenomics and other ‘omics approaches are present in this report. These are driving the emergence of personalized approaches and are often facilitated by artificial intelligence. This advance is promising for patients but requires adaptation to regulating manufacturing, assessing benefit-risk and designing trial, as well as to the economic model of medicine and the healthcare sector.

Radical innovation is being driven, in part, by these ‘omics methods in combination with artificial intelligence. This is allowing systems biology to progress into ever more complex areas such as healthy aging, a new frontier that challenges the very notion of disease.

Finally, the regulatory system’s response to bigger public health threats, such as AMR and climate emergency is also included in this research. AMR is a complex issue that is tackled on multiple fronts, with an One health perspective. This requires regulatory action by fostering innovation in both therapeutics and diagnostic tools, as well as antibiotic stewardship approaches in the healthcare setting and the creation of conducive business models. Some alternatives to antibiotics currently being develop offer a high level of innovation but bring considerable regulatory challenges for the assessment of efficacy and quality of the medicinal products. With regards to the climate emergency, EMA’s eventual role may be unclear, but medicines development and medical research will have to complete adjusting to it over the next 10 years.

In light of this, it is clear that interaction, advice and support between innovative developers and the EMA, such as through ITF meetings, Scientific Advice and Qualification of Novel Methodologies, is fundamental to clarify regulatory requirements and build expertise within the network and should continue to be encouraged in public and by targeting relevant externally funded projects. Multi-stakeholder engagement is considered a key enabler of the Network’s strategic priorities as reflected in the EMAN strategy to 2025. However, innovation and the Regulatory Science Research needs also demand that well-established engagement activities at EMA, originally targeted to specific stakeholder groups, are adapted, as needed, to new areas of work and integrate an earlier multi-stakeholder approach, where possible.

Collaboration across EU agencies and international bodies and knowledge sharing is also essential, as it allows to assimilate lessons learned from different fields or realities and build up the necessary competencies to address the most difficult health problems, which will benefit from an integrated response.

## Data availability statement

The original contributions presented in this study are included in the article/supplementary material, further inquiries can be directed to the corresponding author.

## Author contributions

PH and RH contributed to conception and design of the study. All authors wrote the first draft of the manuscript, contributed to manuscript revision, read, and approved the submitted version.
